# An automated sampling importance resampling procedure for estimating parameter uncertainty

**DOI:** 10.1007/s10928-017-9542-0

**Published:** 2017-09-08

**Authors:** Anne-Gaëlle Dosne, Martin Bergstrand, Mats O. Karlsson

**Affiliations:** 1 Department of Pharmaceutical Biosciences, Uppsala University, Uppsala, Sweden; 2 Pharmetheus, Uppsala, Sweden

**Keywords:** Sampling importance resampling, Parameter uncertainty, Confidence intervals, Asymptotic covariance matrix, Bootstrap, Nonlinear mixed-effects models

## Abstract

Quantifying the uncertainty around endpoints used for decision-making in drug development is essential. In nonlinear mixed-effects models (NLMEM) analysis, this uncertainty is derived from the uncertainty around model parameters. Different methods to assess parameter uncertainty exist, but scrutiny towards their adequacy is low. In a previous publication, sampling importance resampling (SIR) was proposed as a fast and assumption-light method for the estimation of parameter uncertainty. A non-iterative implementation of SIR proved adequate for a set of simple NLMEM, but the choice of SIR settings remained an issue. This issue was alleviated in the present work through the development of an automated, iterative SIR procedure. The new procedure was tested on 25 real data examples covering a wide range of pharmacokinetic and pharmacodynamic NLMEM featuring continuous and categorical endpoints, with up to 39 estimated parameters and varying data richness. SIR led to appropriate results after 3 iterations on average. SIR was also compared with the covariance matrix, bootstrap and stochastic simulations and estimations (SSE). SIR was about 10 times faster than the bootstrap. SIR led to relative standard errors similar to the covariance matrix and SSE. SIR parameter 95% confidence intervals also displayed similar asymmetry to SSE. In conclusion, the automated SIR procedure was successfully applied over a large variety of cases, and its user-friendly implementation in the PsN program enables an efficient estimation of parameter uncertainty in NLMEM.

**Electronic supplementary material:**

The online version of this article (doi:10.1007/s10928-017-9542-0) contains supplementary material, which is available to authorized users.

## Introduction

The added value of modeling and simulation using nonlinear mixed-effects models (NLMEM) for decision-making in drug development has long been advocated and illustrated [1–4]. NLMEM provide a mathematical description of pathophysiological and pharmacological processes, as well as a statistical characterization of the different sources of variability affecting these processes, in particular inter-individual variability. The model structure together with the value of model parameters can be used for a number of applications such as quantifying drug interactions [5], calculating the power of a prospective trial [6], proposing dose regimen adaptations [7] or designing efficient clinical trials [8]. In such applications, the uncertainty around model parameters typically needs to be taken into account. Parameter uncertainty can be quantified using a number of methods, which can lead to different uncertainty estimates. Despite this and contrarily to the scrutiny exercised towards structural and distributional assumptions, the adequacy of uncertainty estimates in NLMEM is rarely inspected. A diagnostic assessing the adequacy of uncertainty estimates in NLMEM was recently developed to start filling this gap [9]. In addition, the Sampling Importance Resampling (SIR) method was proposed to improve the estimation of parameter uncertainty for NLMEM as compared to currently available methods such as the asymptotic variance–covariance matrix and the bootstrap [10]. SIR provides many advantages. From a computational perspective, it is faster than bootstrap. It also does not require any parameter estimation, which contributes to its speed but also avoids common issues due to convergence problems. As bootstrap, SIR does not make any assumptions about the shape of the uncertainty distribution, whereas the covariance matrix is generally used as a multivariate normal distribution. Lastly, SIR can be used with any type of data, unlike the bootstrap which is often limited by design characteristics such as small subgroups or unbalanced sampling.

SIR provides parameter uncertainty, for a given model and set of data, in the form of a defined number *m* of parameter vectors representative of the true and unknown parameter uncertainty distribution. The *m* parameter vectors are obtained by drawing from a set of a defined number *M* (*M* > *m*) of parameter vectors arising from a proposal distribution. When using SIR, three settings need to be chosen: the proposal distribution, the number of samples *M* and the number of resamples *m*. In a previous publication [10], the authors tested different SIR settings on simple NLMEM, and also developed diagnostics to assess whether the chosen settings were appropriate. When using the covariance matrix as a multivariate normal proposal distribution and resampling 1000 parameter vectors out of 5000, SIR was able to recover the true uncertainty in two simulation examples, and led to sensible confidence intervals for three simple, linear pharmacokinetic models. Based on these initial results, in the present work the SIR procedure was extended to an iterative procedure, where the resampled parameters of any given iteration serve as proposal distribution for the next. This iterative procedure has the advantage of more efficiently improving the proposal distribution towards the true uncertainty, as well as enabling direct assessment of the convergence of the SIR procedure. In addition to the improved procedure, the present work also extends the scope of NLMEM on which SIR has been tested from three simple PK models to 25 pharmacokinetic and pharmacodynamic NLMEM featuring continuous and categorical endpoints, with up to 39 estimated parameters and varying degrees of complexity.

The aim of this work was to evaluate the performance of the newly proposed SIR workflow to estimate parameter uncertainty on an array of NLMEM. Parameter uncertainty obtained with SIR was compared to the uncertainty obtained with the commonly used methods based on the covariance matrix, the bootstrap and stochastic simulations and estimations (SSE). 

## Methods

### SIR workflow

SIR was originally developed in Bayesian statistics [11] as a non-iterative procedure. It had been adapted to the estimation of parameter uncertainty in NLMEM [10], where for a given model and set of data, SIR would provide an estimate of uncertainty in the form of a defined number *m* of parameter vectors obtained in the following three steps:

1. Step 1 (sampling): A defined number *M* (*M* > *m*) of parameter vectors were sampled from a multivariate parametric proposal distribution, obtained for example based on the covariance matrix or a limited bootstrap.
2. Step 2 (importance weighting): An “importance ratio”, representing the probability in the true parameter uncertainty distribution, was computed for each of the sampled parameter vectors. According to SIR theory [11], this probability can be approximated by the likelihood of the data given the parameter vector weighted by the likelihood of the parameter vector in the proposal distribution (Eq. 1).
  1 $$IR = \frac{{exp\left( {\frac{ - 1}{2}dOFV} \right)}}{relPDF}$$
  IR is the importance ratio, dOFV is the difference between the objective function value (OFV, equal to minus two times the log-likelihood up to a constant) of the parameter vector and the OFV of the final parameter estimates of the model, and $relPDF$ is the value of the probability density function of the parameter vector relative to the probability density of the final parameter estimates
3. Step 3 (resampling): *m* parameter vectors were resampled from the pool of *M* simulated vectors with probabilities proportional to their importance ratio.

The resampled distribution represents the true uncertainty when the number of samples *M* tends towards infinity [11]. When *M* is finite, SIR results are closer to the true uncertainty than the proposal distribution, but may still differ from the true distribution. How close SIR results are to the true uncertainty depends on both the proposal distribution and *M*: the closer the proposal is to the true uncertainty and the higher *M*, the closer SIR results are to the true distribution. Note that the size of *M* is only important relative to the number of resamples *m,* the true quantity of interest being the ratio *M/m*. *m* is chosen by the user depending on the desired level of precision in uncertainty estimates. For example, if one wants to compute relative standard errors on parameter estimates, *m* can be set to a much lower number than when one wants to compute 95% confidence intervals.

**Fig. 1:**
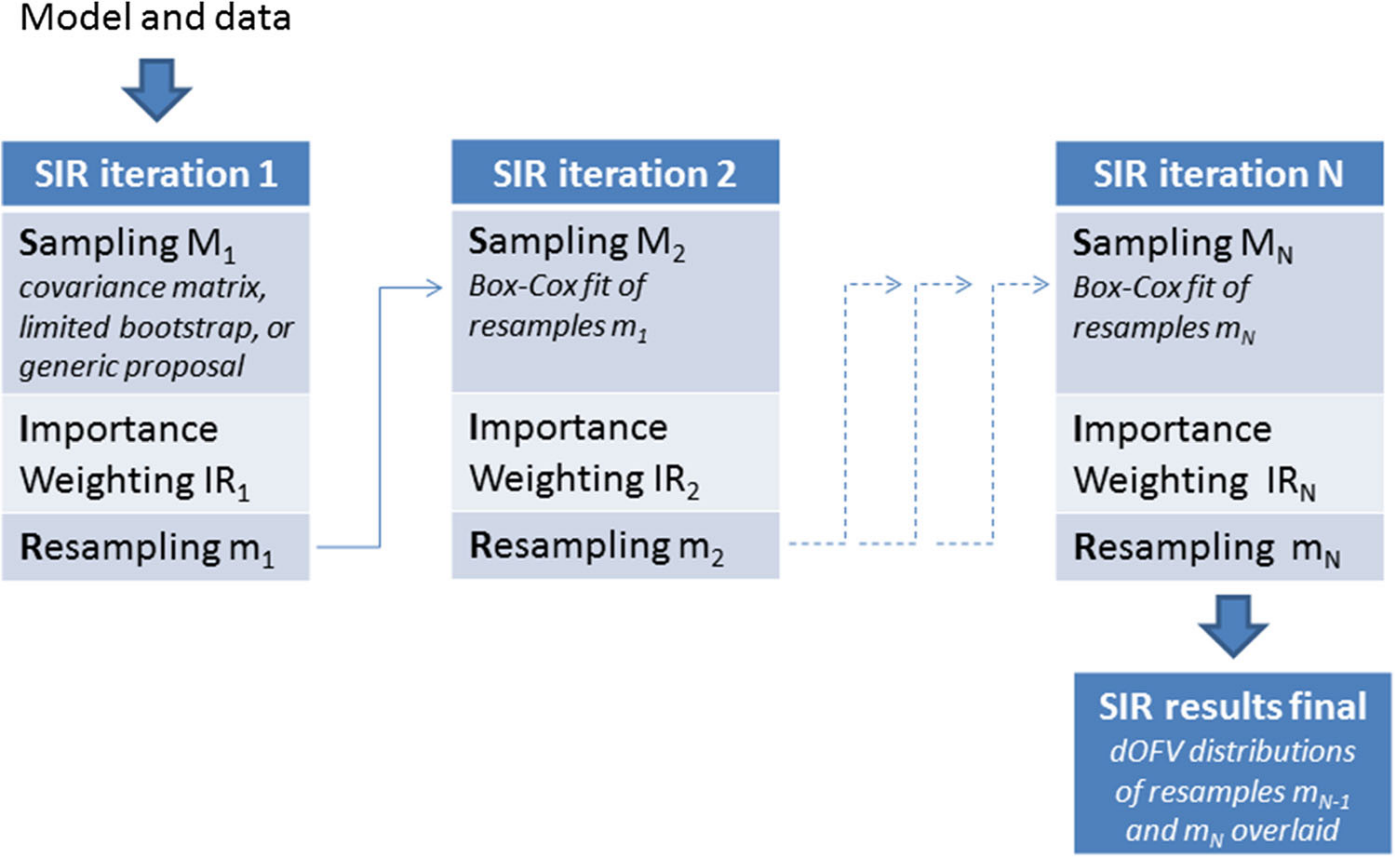
SIR diagnostic plot showing SIR convergence for one of the investigated examples. *Lines* represent the value of the dOFV for each percentile of the proposal distributions (*dotted colored lines*) and the resamples distributions (*solid colored lines*) at each iteration. The *shaded area* represents the resampling noise around the last resamples dOFV distribution. The dOFV resamples distributions of the last two iterations need to be within sampling noise for SIR results to be considered final. The reference Chi square distribution with degrees of freedom equal to the number of estimated parameters is the *grey solid line* and the estimated degrees of freedom for each distribution are displayed in the *bottom right corner*

Given the unknowns around the choice of SIR settings which would lead to the true uncertainty, Dosne et al. [10] proposed two diagnostics to assess how close results of a SIR with user-chosen settings are to the true uncertainty: the dOFV plot and the temporal trends plot. The dOFV plot (exemplified in Fig. 1) is based on the property that if the *m* resampled vectors correspond to the true uncertainty, the distribution of their dOFV, i.e. the difference between the objective function value (OFV, equal to minus two times the log-likelihood up to a constant) of the resampled parameter vector and the OFV of the final parameter estimates of the model, is expected to follow a Chi square distribution with degree of freedom equal or inferior to the number of estimated parameters [12]. If this is the case, i.e. if the dOFV distribution of the SIR resamples lies at or beneath the reference Chi square distribution on the dOFV plot, then the temporal trends plot (as displayed in [10]) is further inspected to ensure that SIR cannot be further improved. If the temporal trends plot shows that the resampling proportion in regions of the proposal displaying high IR does not decrease over the resampling sequence, meaning that SIR cannot be improved by further increasing *M*, SIR settings can be considered appropriate and SIR results considered final. If this is not the case, SIR has to be performed again using a different, typically inflated proposal distribution or an increased *M*, depending on the observed trends in the diagnostics.

The idea of the present work was to improve the SIR procedure to achieve final results in an automated way. To this end, the procedure was extended to an iterative procedure, where the resamples of one iteration were used as the proposal distribution of the next iteration. This was performed by fitting a multivariate Box-Cox distribution to the resamples at each step, and then using this distribution to generate the samples of the next step. The Box-Cox distribution was chosen for two reasons: first, a parametric distribution was required in order to be able to compute the denominator of the importance ratios. Second, contrarily to a multivariate normal distribution, the Box-Cox distribution allows both symmetric and asymmetric distributions through the use of parameter-specific shape parameters. This was expected to better approximate the true uncertainty, notably for variance parameters. Using an iterative procedure also greatly simplified SIR diagnostics, as the resamples distribution was expected to gradually converge to the true uncertainty distribution. SIR results could thus be considered final when no changes are observed between the estimated uncertainty of two consecutive iterations.

The proposal distribution of the first iteration was set to the “sandwich” covariance matrix obtained in NONMEM if available, otherwise to a limited bootstrap (e.g. 200 bootstrap samples). The number of SIR samples and resamples was set as follows: the first three iterations used *M* = 1000 samples and increasing *m* = 200, 400, 500 resamples, while all further iterations used *M* = 2000 samples and *m* = 1000 resamples. This was chosen to maximize efficiency, with high *M/m* ratios and low *m* at the start of the procedure enabling fast improvement over the proposal while keeping runtimes short. From the fourth iteration on, proposals were expected to be relatively close to the true uncertainty, and thus a lower *M/m* ratio with *m* = 1000 thought to enable precise estimation of the uncertainty while minimizing runtime. Note that changes in the number of samples and resamples settings impact SIR efficiency, but they are not expected to impact final SIR results. As such, they can be modified on a case by case basis by the user.

**Fig. 2:**
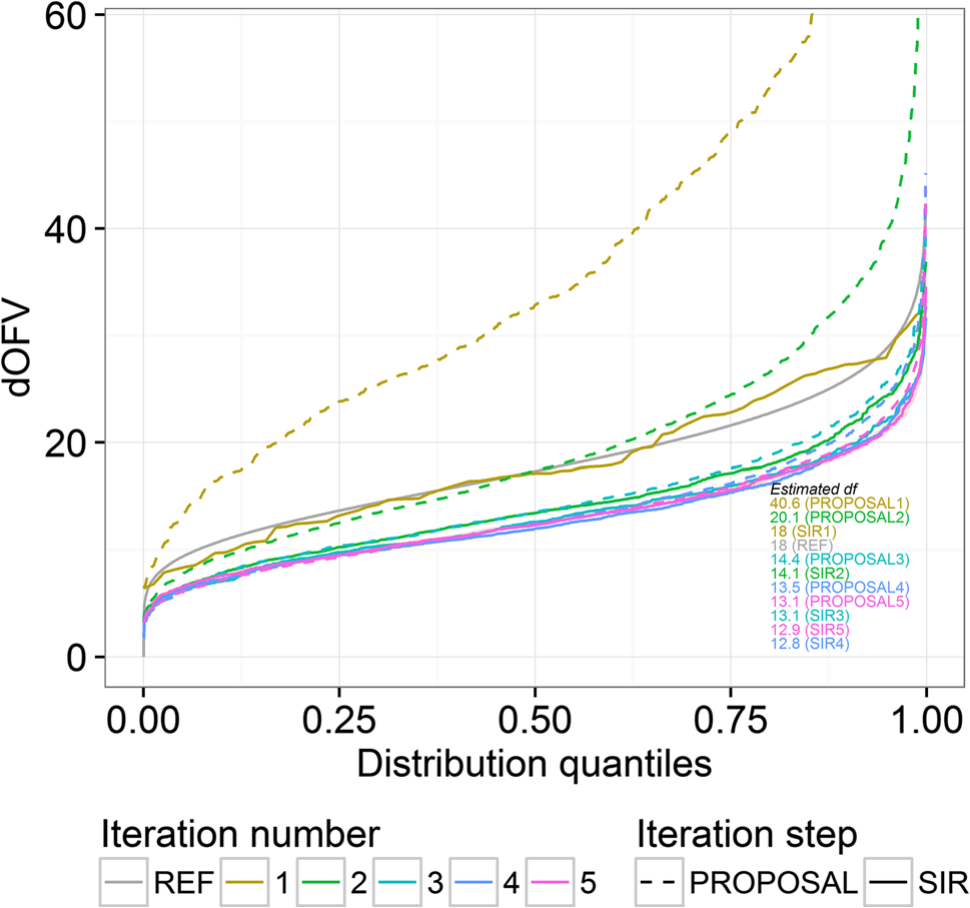
Proposed SIR workflow. To obtain SIR parameter uncertainty for a given model and data, SIR is started using, in order of preference: the covariance matrix, a limited bootstrap (e.g. 200 samples or less). or a generic covariance matrix (e.g. 50% RSE on all parameters) as first proposal distribution. Then, SIR iterations are automatically performed using the resamples of one iteration as proposal distribution of the next, until the dOFV distributions of the resamples of the last 2 iterations are overlaid in the dOFV plot, in which case SIR results are considered final

SIR results were considered final when no changes were observed between the estimated uncertainty of two consecutive iterations. dOFV distributions were used as a surrogate for the estimated uncertainty. SIR convergence was thus assessed based on the dOFV plot, and SIR results were considered final when the dOFV distributions of the resamples of two consecutive iterations were overlaid up to sampling noise. A schematic of the SIR workflow is presented in Fig. 2.

Note that a particular case arises when the dOFV distribution of the first proposal distribution is already below the reference Chi square distribution. Based on the work in [10], this indicates a risk that the initial parameter uncertainty is underestimated. Because it is more difficult for SIR to increase than to decrease uncertainty distributions, SIR should then be restarted using an inflated proposal distribution. In such cases, the proposal was inflated by multiplying all variances and covariances by a single factor, until its dOFV distribution lay above the reference Chi square.

### Testing of the new SIR workflow

The new workflow was tested on 10 pharmacokinetic and 15 pharmacodynamic models, i.e. a total of 25 NLMEM [5, 13–36]. A summary of the models is provided in Table 1, and a more detailed description of each model is available in Table S1 in the Supplementary Material. Around 80% of the modelled endpoints were continuous. The number of estimated model parameters ranged from 1 to 39, with random effects accounting for 0 to 77% of the total number of parameters. Datasets covered sparse to rich data, with an average of 115 individuals, 4076 observations, and 28 observations per individual.

**Table 1:** Summary of the 25 investigated NLMEM models

Model characteristic	Categories/mean value (range)
Type of model	10 PK, 15 PD (total 25)
Type of data	21 continuous, 4 categorical
Number of estimated parameters	15 (1–39)
Proportion of random effects (%)	27 (0–77)
Number of individuals	115 (6–551)
Number of observations	4076 (58–47,784)
Number of observations/individual	28 (1–102)
Estimation method	3 FO, 5 FOCE, 10 FOCEI, 7 LAPLACE

*PK* pharmacokinetic, *PD* pharmacodynamic, *FO* first-order, *FOCE* first-order conditional estimation, *FOCEI* first-order conditional estimation with interaction

The evaluation of the iterative SIR procedure was based on the number of iterations needed until stabilization and on the degree of freedom of the dOFV distribution obtained at stabilization, which is a marker of the adequacy of the uncertainty estimates and should be equal to or lower than the number of estimated parameters [10]. Typical and atypical behaviors were reported and analyzed.

### SIR sensitivity to initial proposal distribution

The influence of the initial proposal distribution was investigated by performing the iterative procedure as described above but using a generic covariance matrix as initial proposal distribution. This generic SIR was compared to the informed SIR (starting from the covariance matrix or a limited bootstrap) in order to assess whether SIR was robust to the initial proposal distribution and the extent of the loss in runtime when starting from less informed proposal distributions. The generic covariance matrix was set to a multivariate normal distribution with 30% relative standard errors (RSE) on fixed effects, 50% RSE on inter-individual and inter-occasion variabilities, 10% RSE on residual variabilities, and no correlations between any of the parameter uncertainties. Except for the initial proposal distribution, the workflow remained as presented in Fig. 2. The number of iterations until stabilization, the runtime, the final parameter RSE and the final degree of freedom were contrasted between the generic and the informed SIR.

### Comparison of SIR with other methods for parameter uncertainty

Finally, the uncertainty obtained with SIR was compared to the uncertainty obtained with three other methods: the covariance matrix, case bootstrap, and SSE. The covariance matrix was obtained from the sandwich estimator in NONMEM if possible. The bootstrapped datasets were constructed by resampling individual data vectors with replacement. Bootstrap uncertainty estimates were obtained based on all available bootstrapped parameter estimates, regardless of the termination status of the estimation. For the SSE, also known as parametric bootstrap, datasets were simulated based on the final model (using final parameters estimates and the design of the original dataset), and parameters were re-estimated on the simulated datasets using the same model. As for case bootstrap, SSE uncertainty estimates were obtained based on all available parameter estimates regardless of the termination status. Parameter uncertainty was based on 1000 samples for all methods. Following metrics were compared: runtimes, RSE, relative widths and relative asymmetry of the parameters’ 95% confidence intervals (95% CI). The relative widths of the confidence intervals were calculated as the distance between the confidence interval’s upper and lower bounds, divided by the final parameter estimate of the original dataset. Asymmetry was quantified by the ratio of the distance between the confidence interval’s upper bound and the median, divided by the distance between the confidence interval’s lower bound and the median. Runtime comparisons were performed between SIR and bootstrap based on the ratio between the time 7000 likelihood evaluations were expected to take (as performed during the SIR procedure, i.e. using MAXEVAL = 0 in $EST in NONMEM) and the time 1000 likelihood estimations were expected to take (as performed during the bootstrap, i.e. using MAXEVAL = 9999 in $EST in NONMEM). As it was not known in advance how many iterations SIR would require, the benchmark used for SIR consisted of the runtime needed for 5 iterations, which was considered reasonable for the majority of models. The absolute runtime of each procedure could not be used due to its dependency on cluster load, user settings and parallelization processes. Runtime comparisons with the covariance matrix or the SSE were not performed as the computation of the covariance matrix was expected to be markedly faster than likelihood evaluations or estimations in the vast majority of cases, and the SSE was expected to have runtimes similar to bootstrap.

### Software

The iterative SIR procedure was implemented as an automated function in the modeling support tool PsN [37]. NONMEM 7.3 [38] and PsN 4.5.0 and above were used to perform all SIR, bootstraps and SSE. RStudio 0.98 using R 3.1.2 [39] was used for graphical output.

## Results

### Performance of the new proposed SIR procedure

The asymptotic covariance matrix was available to be used as proposal distribution for SIR for 20 models. For 5 models, the covariance matrix could not be obtained, so SIR was performed using a limited bootstrap as initial proposal distribution. Inflation of the covariance matrix was needed for 9 out of 20 models, as the dOFV distribution of the covariance matrix appeared partly or fully below the reference Chi square distribution. An inflation of all variances by 1.5 was sufficient to correct the underestimation for six models, but inflation of 2 and 3 were used for the three remaining models.

**Fig. 3:**
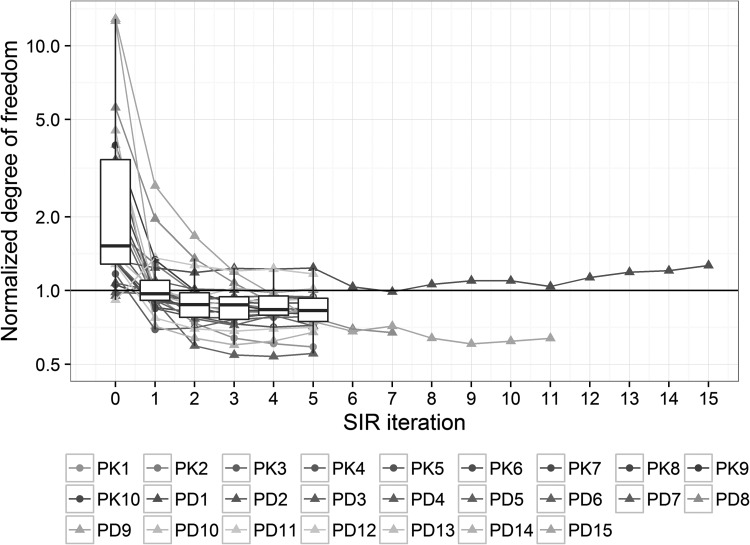
Convergence of the informed SIR over the 25 investigated models as represented by the estimated degree of freedom of the SIR resamples distribution at each iteration, normalized by the total number of estimated parameters of each model. The normalized degree of freedom at the 0th iteration is the degree of freedom of the informed proposal distribution (covariance matrix or limited bootstrap). *Boxplots* represent the median, first and third quartiles of the degree of freedom during the proposed iterative procedure until the 5th iteration, when most of the models had converged

Convergence at the end of the iterative SIR procedure was assessed visually based on the dOFV distribution plot. Other diagnostic plots were also inspected. An example of dOFV plot where SIR has converged is provided in Fig. 1. An example of dOFV plot where SIR requires inflation is provided in Figure S1 in the Supplementary Material. SIR convergence was achieved after 3 iterations on average, with only three models needing more than 5 iterations to converge. Two of these models converged after 7 (PD8) and 11 (PD15) iterations respectively. The last model (PD1) kept oscillating around a degree of freedom above the total number of parameters (around 29, with 23 estimated parameters). The degree of freedom (df) also stabilized slightly above the total number of parameters for PD11 (df = 7, with 6 estimated parameters). SIR convergence for the different models is illustrated in Fig. 3, which displays the estimated degree of freedom of the SIR resamples distribution at each iteration, normalized by the total number of estimated parameters of the model. The degree of freedom estimated at the 0th iteration corresponds to the degree of freedom of the initial proposal distribution, i.e. the covariance matrix or the limited bootstrap. The estimated degrees of freedom of the final SIR distributions were on average 20% lower than the total number of estimated parameters [range (−40%; +30%)]. The initial proposal distributions (model’s covariance matrix or limited bootstrap) appeared different from the true uncertainty, with degrees of freedom higher than the number of estimated parameters: median degrees of freedom were 1.4-fold higher than the number of parameters for the covariance matrices, and 4-fold higher for the limited bootstraps.

### SIR sensitivity to initial proposal distribution

**Fig. 4:**
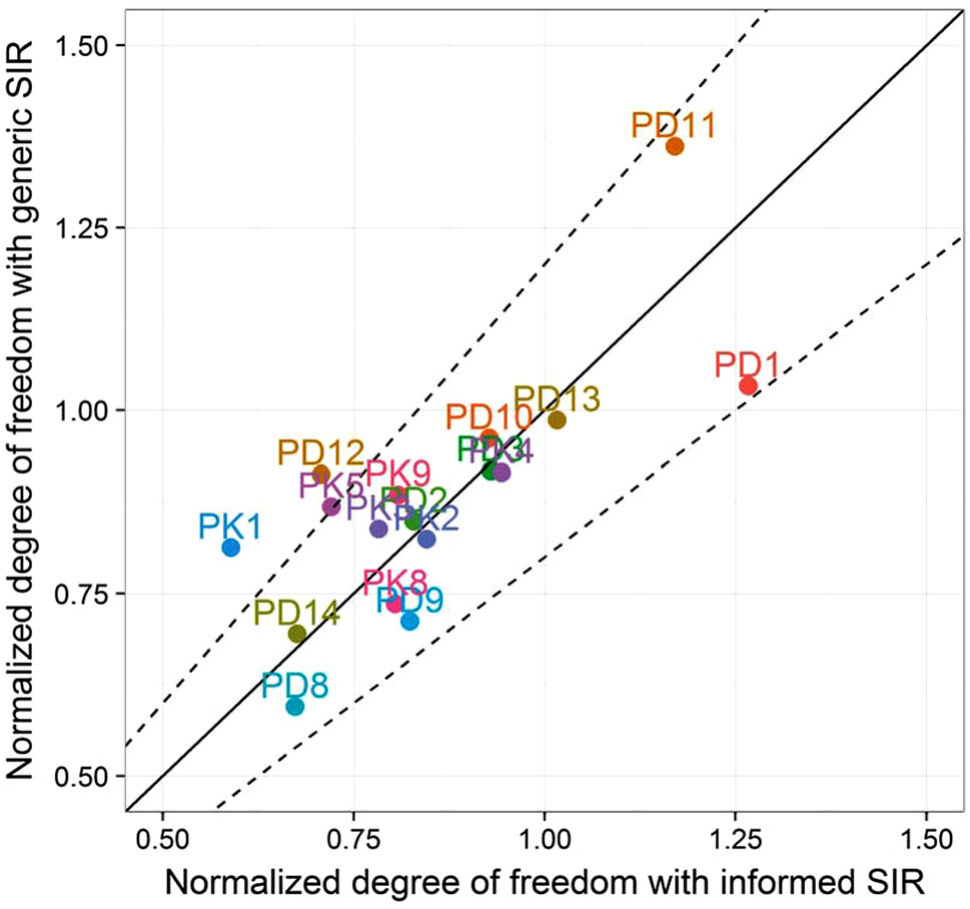
Normalized degree of freedom of the SIR resamples distribution at stabilization for the generic SIR (*y*-axis) and for the informed SIR (*x*-axis) for the 17 models for which results from both SIR were available. The *full black line* is the identity line and the *dashed lines* represent deviations of 20% from the identity line

Results obtained with the SIR procedure starting from a generic multivariate normal distribution were available for 17 out of the 25 models. Regarding runtime, as expected it took more iterations for SIR to converge when starting from the generic proposal distribution: 8 iterations were needed on average for the generic SIR to converge, versus 3 iterations for the informed SIR. In theory, final SIR results should not be sensitive to the initial proposal distribution, as long as the procedure is pursued with sufficient number of iterations and/or sufficient number of samples *M*. RSE obtained after SIR convergence with the two methods changed by less than 5% on average and were generally comprised within 20% of the value obtained with the informed SIR (results not shown). This illustrated the robustness of SIR towards its proposal distribution. The closeness of the estimated degrees of freedom at stabilization between the generic and the informed SIR, which differed by less than 5% on average [range (−19%; +40%), Fig. 4] further supported this finding for most models. The PK1 model showed an 8-point difference in degrees of freedom between the two SIR. The lower adequacy of the generic initial proposal distribution was also reflected in the estimated degrees of freedom at start of the SIR procedure. The degrees of freedom were around 25-fold higher than the number of estimated parameters for the generic SIR, versus 1.4 to 4-fold with the informed SIR.

### Comparison of SIR with other methods for parameter uncertainty

Estimates of parameter uncertainty using the covariance matrix, the bootstrap and the SSE were available for 20, 19 and 16 models respectively. In terms of runtime, SIR was on average 10 times faster than bootstrap. SIR was faster than the bootstrap for all models except for two models run with the FO method. The greatest runtime gain was observed for an epilepsy model estimated with the LAPLACE method, for which one estimation of the likelihood took 2000 times longer than one evaluation of the likelihood. Note that SIR runtime gains calculated as detailed in the Methods section are expected to be overestimated, as the processing time between the different iterations was not taken into account.

**Fig. 5:**
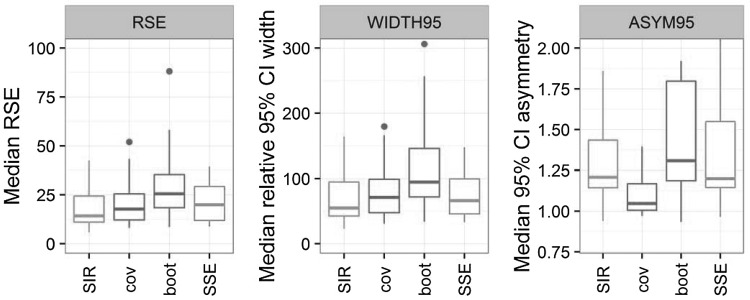
Distribution of the median (over all parameters) RSE, 95% CI width (WIDTH95) and asymmetry (ASYM95) for all models by uncertainty method: SIR, covariance matrix (cov), bootstrap (boot) and SSE

Differences between parameter uncertainties obtained with the four methods were highly model- and parameter-dependent. To be able to investigate general ten-den-cies, the median value of each uncertainty metric (RSE and width and asymmetry of the 95% CI) was computed for each model, over all of its estimated parameters. Methods were only compared to one another, as the true parameter uncertainty of the real datasets was unknown. Median RSE over all model parameters were similar between all methods but the bootstrap, which showed higher RSE (Fig. 5, left panel). This was also reflected in the relative width of the 95% CI (normalized by the parameter value): the covariance matrix and SSE led to similar CI widths, whereas SIR led to slightly narrower CI and the bootstrap to much wider CI (Fig. 5, middle panel). 95% CI with SIR was 15% narrower than with SSE and 40% narrower than with the bootstrap. In terms of asymmetry, SIR was close to SSE, with median asymmetry values around 1.2 (Fig. 5, right panel). This meant that the upper bounds of the CI were 1.2-fold further away from the median than the lower bounds. The bootstrap displayed the highest asymmetry (median at 1.3), and the covariance matrix the smallest (median below 1.1).

Differences in the estimated parameter uncertainty between the four methods were also reflected in the estimated degrees of freedom. Median normalized degrees of freedom were closest to 1 for the SSE (df = 1.1), followed by SIR (df = 0.8), the covariance matrix (df = 1.4) and finally bootstrap (df = 1.6).

## Discussion

To summarize, the iterative SIR procedure starting from the covariance matrix or a limited bootstrap was satisfactory for 22 out of the 25 models investigated. As indicated from the dOFV plot, the proposal required inflation in about half the cases to increase SIR efficiency in the presence of misspecified narrow RSE. As SIR seemed relatively robust to the choice of initial proposal distribution, it is recommended to use an informed proposal distribution if available, as this will considerably decrease runtimes. SIR led to median RSE and CI widths comparable to the covariance matrix and SSE, and smaller than the bootstrap. The asymmetry of the CI with SIR was similar to the asymmetry with the SSE and was lower than the asymmetry with the bootstrap. SIR seemed to perform better than the other methods based on the estimated degrees of freedom, as higher values indicate that parameter vectors of the uncertainty distribution are unlikely based on the model and data. Details of these different aspects are discussed below.

### Performance of the new proposed SIR procedure

Starting the iterative SIR procedure from the covariance matrix required inflation by factors of 1.5 and above for half of the models, but no inflation was necessary when starting from a limited bootstrap. This underestimation was to relate to the use of a symmetric multivariate normal distribution when computing the proposal distribution based on the covariance matrix. Symmetric distributions are known to be unlikely to hold true for random effects, which are expected to follow asymmetric right-skewed distributions bounded by 0 in their lower tail, such as inverse Wishart distributions. However, in order to compute the probability density function of the full uncertainty distribution (i.e. including all fixed and random effects and taking potential correlations into account), it was not possible to use different distributions for fixed and random effects. It was expected that random effects would as a consequence see their distribution shifted downwards compared to a corresponding inverse Wishart distribution with identical variance. This was only the case for the first covariance matrix-based iteration, as a more flexible distribution, the Box-Cox distribution, was used for all subsequent SIR iterations and when starting from a limited bootstrap. The implemented multivariate Box-Cox distribution included parameter-specific shape parameters governing the skewness of each parameter’s distribution. Estimating a shape parameter for each model parameter enabled proposal distributions to be symmetric for fixed effects but right-skewed for random effects, as is often observed. An inverse Wishart distribution would have been less flexible than the Box-Cox distribution, as the variance and the skewness of the inverse Wishart distribution are defined by a single parameter, which forces the distribution to be asymmetric when its variance is high. The shape parameters of the Box-Cox distribution were estimated on the set of parameter vectors obtained by bootstrap or by SIR resampling. Using a Box-Cox distribution was not possible when starting directly from the covariance matrix, as no parameter vectors were available to estimate the shape parameters. Further improving the automated SIR procedure by starting from a multivariate Box-Cox distribution with fixed shape parameters, for example 1 (symmetric distributions) for fixed effects and 0 (right skewed distributions, equivalent to log-normal) for random effects, could be considered. This could potentially decrease the need for inflation of the proposal distribution. Also, an automated check could be implemented after the first iteration to detect whether the proposal dOFV distribution is partly or fully below the Chi square distribution. If this is the case, the proposal could be automatically inflated until it is fully above the Chi square distribution prior to starting the iterative SIR procedure.

Limitations of the Box-Cox distribution as a parametric approximation of a set of parameter vectors were sometimes apparent, for example when comparing the degree of freedom at the first iteration when starting from the limited bootstrap to the degree of freedom obtained with the full bootstrap. If the Box-Cox distribution were a good approximation of the (nonparametric) bootstrap parameter vectors, both degrees of freedom should be similar. The degrees of freedom using the Box-Cox distribution was however on average 3 times higher than the degrees of freedom using the full bootstrap, confirming the limitation of the Box-Cox distribution to fully capture the uncertainty reflected in sets of parameter vectors. However, the limitations of the Box-Cox distribution did not limit SIR performance as long as the *M/m* ratios were high enough. The use of even more flexible multivariate distributions than the Box-Cox could be envisaged to further increase SIR efficiency. An alternative may also be to perform the sampling in a less random manner, in order to guarantee a better representation of the proposal [40].

Another characteristic of multivariate distributions is the correlation structure they imply. For the multivariate normal distribution, correlations were assumed to be linear; in the implementation of the multivariate Box-Cox distribution, they were assumed to be linear on the Box-Cox transformed scale, resulting in fixed shape-dependent correlations on the untransformed scale. Similar to the problem with too narrow proposal distributions, the SIR procedure will have difficulties to correct misspecified correlations shapes when correlations are high, as these restrict the parameter space investigated by SIR. Half of the models showed one or more correlations greater than 0.8 in their proposal distributions. These correlations decreased below 0.8 in approximately half of the cases, showing that SIR was to some extent able to decrease too high correlations. On the other hand, SIR was also able to pick up correlations, most notably when starting from the generic proposal distribution, for which correlations were set to 0. The occurrence of problematic cases due to misspecified correlations seemed low in the investigated models, however experience with other models outside the scope of this work has confirmed constrained correlations structures to be a potential issue for SIR if misspecified. A default cap on correlations during the iterative procedure could be beneficial to avoid any undue restriction of the parameter space explored by SIR.

An automated, numerical stop of the SIR procedure based on the stabilization of the degree of freedom instead of the visual inspection of dOFV distributions was not considered, notably because the required tolerance towards between-iteration variations of the degree of freedom could not easily be determined.

The degree of freedom of the final SIR resamples distribution was on average around 80% of the total number of estimated parameters (minimum at 60% for one model, and between 60 and 80% for 10 models). This seemed reasonable, as for NLMEM the degree of freedom is unknown but expected to be at or below the number of estimated parameters due to restrictions in the parameter space (such as the positive domain for variances, or given by physiological boundaries). Potential explanations for lower degrees of freedom are a high proportion of random effects [41], as variances might contribute to less than a full degree of freedom, as well as small dataset sizes, for which the properties of the likelihood ratio test are not always respected [42]. Efforts to link the final degree of freedom to model characteristics were not successful: no correlation between the proportion of random effects, sample size (number of individuals, observations or observations per individual) and degree of freedom could be established.

### SIR sensitivity to initial proposal distribution

SIR results obtained when starting from a generic proposal distribution with RSE of 30% on fixed effects, 50% on random effects and 10% on residual errors were in general very similar to those obtained using the informed proposal. Given the similarity of the results between the generic and informed SIR for all models but one and the approximated 2-fold loss in runtime with the generic SIR, it is recommended to use an informed proposal as initial distribution.

### Special cases

Three models showed atypical behavior when performing the SIR procedure. The first model, PD1 (Likert pain score model based on Poisson distributions with Markovian elements), showed unstable degrees of freedom and RSE up until 15 SIR iterations. For this model the covariance matrix could not be obtained, and bootstrap and SSE results highlighted major estimations issues (only 1% of the minimizations were successful, and only 5% of the datasets led to parameter estimates different from the initial estimates). This highlights a limitation of SIR when the likelihood cannot be reliably evaluated. However, such a limitation is often shared between uncertainty estimation methods.

The second model, PD11 (time to event model for conversion to sinus rhythm in acute atrial fibrillation), stabilized at a degree of freedom greater than the total number of parameters. This model also displayed estimation issues during the bootstrap. One of the parameters, a threshold value for a change in hazard, was shown to be the source of these problems: fixing it to its estimated value lead to more sensible SIR results with a degree of freedom of 4.5 for 5 estimated parameters.

The last atypical model was PK1, a 31-parameter model of parent and metabolite PK data displaying a degree of freedom 8 points higher with the generic SIR than the informed SIR at stabilization. This translated into higher RSE for some variance parameters, notably the inter-individual variability on the central volume which RSE increased from 34% (informed SIR) to 56% (generic SIR). An inflation of the proposal of the informed SIR corrected the problem, and led to identical degrees of freedom at stabilization for both SIR. The fact that the underestimation of the uncertainty of some variance parameters was not visible in the diagnostic plots of the informed SIR remains to be fully understood. It could be linked to the high number of estimated parameters, which could diminish the power of parameter-specific diagnostics to detect trends based on single parameters.

Two models highlighted interesting SIR features. The first model, PD15 (diabetes model linking insulin, glucose and weight), was found to be at a local minimum during the SIR procedure: multiple sets of parameter vectors sampled from the covariance matrix were found to have lower OFV than the final estimates. The estimation was thus restarted using the vector with the lowest OFV as initial estimates, and SIR was performed on this model. The possibility of finding local minima is thus another advantage of SIR, as it evaluates a high number of parameter vectors spanning a wide parameter space. A warning is outputted if negative dOFV are found during the SIR procedure, so that the local minimum can be addressed. Lastly, PK8 (physiologically based PK model) confirmed the validity of SIR for models with frequentist priors. The uncertainty of parameters associated with priors is known to be underestimated with methods like bootstrap or SSE, which was observed here: both these methods estimated RSE below 10%, whereas SIR RSE were as high as 50% for these parameters.

### Performance of the different methods for parameter uncertainty

The estimated parameter uncertainty differed between SIR, the covariance matrix, bootstrap and SSE. The SSE is sometimes considered the true uncertainty. However, this is only true under no model misspecification, no estimation issues and no design limitation (e.g. dose adaptation based on the modelled outcome, if it is not taken into account in dataset simulation), which is why it was not considered as a reference here. Nevertheless, SIR provided median RSE and CI widths relatively similar to the covariance matrix and SSE, which supported the validity of the developed procedure for uncertainty estimation. The fact that SIR provided asymmetry estimates close to SSE showed its improvement over the covariance matrix, which performed well in terms of uncertainty magnitude (RSE ad CI width) but not symmetry. Bootstrap also performed well describing the shape of the uncertainty, but led to uncertainty magnitudes markedly higher than the other methods, potentially overestimating variability due to suboptimal stratification. A more detailed analysis of the performance of each estimation method will now be provided.

**Fig. 6:**
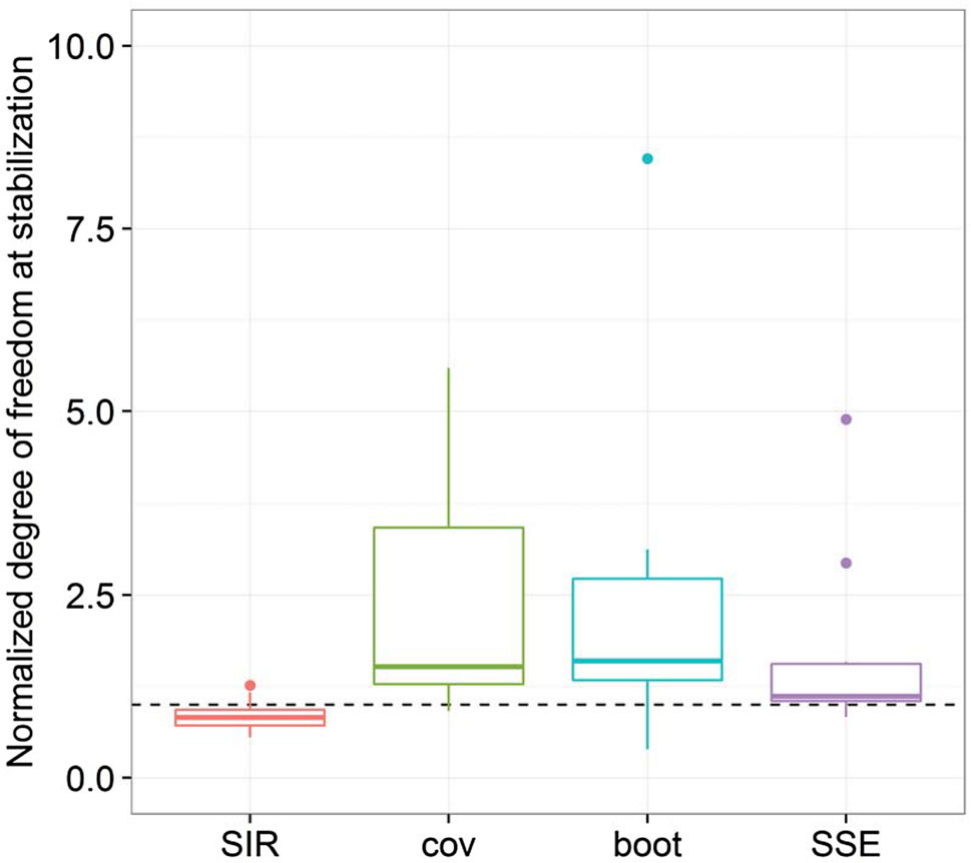
Normalized degree of freedom by uncertainty method: SIR, covariance matrix (cov), bootstrap (boot) and SSE. The *dashed horizontal line* corresponds to a degree of freedom equal to the total number of estimated parameters

Using the covariance matrix to compute parameter uncertainty led to RSE and CI widths slightly higher than SIR (Fig. 5). This might at first seem counterintuitive, as the covariance matrix often had to be inflated to be used as a wide enough proposal for SIR. The need for inflation was however mainly due to the lack of asymmetry, which impacts the CI bounds but not necessarily the RSE, and thus it is not surprising that uncertainty magnitude did not differ much between the two methods. The quasi-absence of asymmetry was expected from using a multivariate normal distribution, which per definition leads to symmetric confidence intervals. Some asymmetry could nevertheless be present due to the parameter boundaries implemented in the models, which led to a truncation of the values outside the specified boundaries. Regarding overall adequacy, the normalized degrees of freedom of the covariance matrix was mostly between 1 and 4 (Fig. 6). Degrees of freedom furthest away from the total number of model parameters were observed for models with poor properties of the covariance matrix (condition number >10^5^ for PD15, Hessian but not sandwich estimator available for PK9), with low number of individuals (PD10), or estimated using the FO method (PK2 and PK7).

Of all uncertainty methods, bootstrap led to the largest RSE and CI widths (Fig. 5). Bootstrap provided high asymmetry in uncertainty distributions, as shown by median asymmetry estimates higher than with the other methods (Fig. 5). The adequacy of the bootstrap as quantified by the degree of freedom seemed suboptimal, with degrees of freedom mostly similar to the covariance matrix (mostly between 1 and 3-fold the total number of parameters, Fig. 6) but less variable. Models that had displayed degrees of freedom furthest away from the number of parameters with the covariance matrix also did so with the bootstrap. This was not unexpected as the covariance matrix is known to be a less good approximation of uncertainty under limitations that can also be problematic for bootstrap, such as high nonlinearity and low sample sizes. Models with bootstrap degrees of freedom furthest away from the number of parameters (PK1, PK5, PD8, PD9 and PD14) displayed estimation problems, with only half of the samples terminating successfully, final 0-gradients and/or estimates near boundaries in at least 20% of the bootstrap samples. These models featured a high number of parameters, highly nonlinear processes and/or correlated parameters. One might argue that the selected setting of retaining all bootstrap estimates regardless of termination status was unfavorable for bootstrap performance. However, in many cases this has not been shown to greatly impact uncertainty estimation [43]. As this work was primarily aimed at evaluating SIR over a complex range on NLMEM and not at providing an exhaustive comparison between SIR and bootstrap, bootstrap settings were chosen for the sake of simplicity and homogeneity of the investigations. Further discussion on handling stratification and estimation problems with the bootstrap will not be touched upon here.

RSE and CI widths based on SSE (i.e. parametric bootstrap) were similar to the covariance matrix and SIR, but lower than bootstrap (Fig. 5). The median asymmetry was close to the asymmetry of SIR, but SSE led to more extreme values. SSE adequacy based on the degrees of freedom was better than the bootstrap and the covariance matrix, with the SSE degree of freedom within 20% of the number of parameters for half of the models. In previous work with much simpler models [9], the degree of freedom using SSE had been found to equal the number of parameters. This was not the case here (Fig. 6). Estimation issues were thought to be responsible for degrees of freedom not being at or below the number of parameters, as many models utilized here were highly complex. Models displaying issues with bootstrap were expected to show issues with SSE, as both methods are based on estimation of datasets assumed to be representative of the population. They should thus lead to results similar up to model misspecification, which is potentially present in the bootstrap but not in the SSE. The adequacy of the SSE uncertainty was indeed better than the bootstrap in 75% of cases. Four models displayed however higher degrees of freedom with the SSE than with the bootstrap (PK9, PD3, PD1, PD11). Other particularly inadequate SSEs were linked to important estimation problems (PK2 with FO, PD5).

## Conclusion

In conclusion, the automated SIR procedure was successfully applied over a large variety of cases, and its user-friendly implementation in the PsN program enables an efficient estimation of parameter uncertainty in NLMEM.

## Electronic supplementary material

Below is the link to the electronic supplementary material.

Supplementary material 1 (DOCX 29 kb)

Supplementary material 2 (DOCX 140 kb)

Supplementary material 3 (DOCX 617 kb)
