# Supplementary material for: An automated sampling importance resampling procedure for estimating parameter uncertainty
Source: J Pharmacokinet Pharmacodyn. 2017 Sep 8;44(6):509–20. doi: 10.1007/s10928-017-9542-0 (PMC5686280; doi:10.1007/s10928-017-9542-0)
Supplement: Supplementary file 1 — Supplementary material 1 (DOCX 29 kb) [file 10928_2017_9542_MOESM1_ESM.docx]

### Supplementary material 1

### **Table S1.** Description of the investigated models

| **Model name** | **Description** | **Model type** | **Ref** | **Data type** | **N**  **obs** | **N**  **ID** | **N**  **obs/**  **ID** | **N**  **par** | **N**  **IIV** | **N**  **COV** | **N**  **IOV** | **N**  **RUV** |
| --- | --- | --- | --- | --- | --- | --- | --- | --- | --- | --- | --- | --- |
|  |  |  |  |  |  |  |  |  |  |  |  |  |
| **PK1** | Single dose bedaquiline and metabolite PK in healthy volunteers (interaction study) | Oral 3-CMT and 2-CMT parent and metabolite PK with transit CMT absorption | 5 | cont | 1044 | 16 | 65 | 31 | 9 | 1 | 2 | 2 |
| **PK2** | Semiphysiological PK model for artemisinin in healthy subjects | Oral 2-CMT PK model with autoinduction and saturable first-pass hepatic extraction | 13 | cont | 475 | 23 | 21 | 11 | 1 | 0 | 2 | 1 |
| **PK3** | Influence of leukemic cell burden on daunorubicin PK in AML patients | i.v. 2-CMT PK with covariates | 14 | cont | 112 | 41 | 3 | 9 | 3 | 1 | 0 | 1 |
| **PK4** | Gentamicin PK in cancer patients | i.v. 2-CMT PK with covariates | 15 | cont | 574 | 210 | 3 | 11 | 2 | 0 | 0 | 2 |
| **PK5** | Midazolam PK in morbidly obese patients before and after bariatric surgery | Oral 3-CMT PK model with transit absorption and covariates | 16 | cont | 808 | 20 | 40 | 18 | 4 | 0 | 0 | 1 |
| **PK6** | Gentamicin PK in preterm and term neonates | i.v. 3-CMT PK with covariates | 17 | cont | 894 | 62 | 14 | 13 | 2 | 0 | 0 | 2 |
| **PK7** | Moxonidine PK in patients with congestive heart failure | Oral 1-CMT PK with covariates | 18 | cont | 1022 | 74 | 14 | 13 | 7 | 1 | 3 | 1 |
| **PK8** | Whole-Body Physiologically-Based PK Model for Colistin and its prodrug in rat | i.v. 14-CMT PBPK model with priors | 19 | cont | 93 | 6 | 16 | 19 | 3 | 0 | 0 | 4 |
| **PK9** | PK of Edoxaban and Its Main Metabolite in a Dedicated Renal Impairment Study | 2- and 1-CMT parent-metabolite PK models with delayed absorption and elimination with covariates | 20 | cont | 813 | 32 | 25 | 22 | 9 | 1 | 0 | 4 |
| **PK10** | PK of Miltefosine in Old World cutaneous leishmaniasis patients | Oral 2-CMT PK model | 21 | cont | 350 | 31 | 11 | 11 | 5 | 1 | 0 | 1 |

**Table S1.** Description of investigated models *(continued)*

| **Model name** | **Description** | **Model type** | **Ref** | **Data type** | **N**  **obs** | **N**  **ID** | **N**  **obs/**  **ID** | **N**  **par** | **N**  **IIV** | **N**  **COV** | **N**  **IOV** | **N**  **RUV** |
| --- | --- | --- | --- | --- | --- | --- | --- | --- | --- | --- | --- | --- |
|  |  |  |  |  |  |  |  |  |  |  |  |  |
| **PD1** | Likert Pain Score Modeling | Truncated generalized Poisson distribution with Markovian transition probability inflation | 22 | cat | 22492 | 231 | 97 | 23 | 12 | 3 | 0 | 0 |
| **PD2** | PK/PD models for T cell-depletion by a monoclonal antibody in patients with multiple sclerosis | Proportional odds model | 23 | cat | 665 | 47 | 14 | 3 | 1 | 0 | 0 | 0 |
| **PD3** | Kinetic-PD tumor-growth inhibition model of drug effect and resistance | 1-CMT tumor growth model (different data than in publication) | 24 | cont | 1248 | 260 | 5 | 8 | 3 | 0 | 0 | 1 |
| **PD4** | Modeling of seizure count in epileptic patients | Zero-inflated negative binomial model with Markovian features | 25 | cat | 47784 | 551 | 87 | 10 | 6 | 2 | 0 | 0 |
| **PD5** | Models for Plasma Glucose, HbA1c, and Hemoglobin Interrelationships in Patients withType 2 Diabetes Following Tesaglitazar Treatment | Oral PK 1-CMT, indirect response model and lifespan-type model with transit CMT | 26 | cont | 8698 | 412 | 21 | 27 | 9 | 0 | 0 | 3 |
| **PD6** | Model for epileptic seizures in patients undergoing antiepileptic drug withdrawal | Repeated Time-To-Event model with Weibull hazard | 27 | cont | 430 | 111 | 4 | 8 | 1 | 0 | 0 | 0 |
| **PD7** | Cox-Proportional hazard model | Cox-Proportional hazard model | 28 | cat | 84 | 42 | 2 | 1 | 0 | 0 | 0 | 0 |
|  |  |  |  |  |  |  |  |  |  |  |  |  |
| **PD8** | Integrated Model for Glucose and Insulin Regulation in Healthy Volunteers Patients Following Intravenous Glucose Provocations | Semi-mechanistic 2-CMT models for glucose and insulin with feedback mechanisms | 29 | cont | 6225 | 70 | 89 | 39 | 19 | 6 | 0 | 4 |

| **Table S1.** Description of investigated models *(continued)* | | | | | | | | | | | | |
| --- | --- | --- | --- | --- | --- | --- | --- | --- | --- | --- | --- | --- |
| **Model name** | **Description** | **Model type** | **Ref** | **Data type** | **N**  **obs** | **N**  **ID** | **N**  **obs/**  **ID** | **N**  **par** | **N**  **IIV** | **N**  **COV** | **N**  **IOV** | **N**  **RUV** |
|  |  |  |  |  |  |  |  |  |  |  |  |  |
| **PD9** | Furosemide PK in congestive heart failure patients | Oral PK 1–CMT with transit absorption | 30 | cont | 170 | 43 | 4 | 11 | 3 | 0 | 0 | 1 |
| **PD10** | Multistate Tuberculosis Model in Patients With Rifampicin-Treated Pulmonary Tuberculosis | Fixed 1-CMT oral PK model with transit absorption and autoinduction coupled to 3-state bacterial growth PD model | 31 | cont | 400 | 23 | 17 | 6 | 1 | 0 | 0 | 2 |
| **PD11** | Characterizing time to conversion to sinus rhythm under digoxin and placebo in acute atrial fibrillation | PK coupled with PD time-to-event exponential model with covariates | 32 | cont | 1168 | 227 | 5 | 6 | 0 | 0 | 0 | 0 |
| **PD12** | Integrated Model for Glucose and Insulin to describe oral glucose tolerance tests in type 2 diabetic patients | Semi-mechanistic 2-CMT models for glucose and insulin with feedback mechanisms | 33 | cont | 4290 | 42 | 102 | 14 | 5 | 1 | 1 | 1 |
| **PD13** | PKPD analysis of the effect digoxin on heart rate in patients with acute atrial fibrillation | Fixed i.v. 2-CMT model with direct response model | 34 | cont | 1168 | 227 | 5 | 2 | 0 | 0 | 0 | 0 |
| **PD14** | Bacterial growth in rats treated with rifampicin, based on multistate tuberculosis model | Fixed 1-CMT oral PK model 3-state bacterial growth PD model | 35 | cont | 58 | 60 | 1 | 11 | 0 | 0 | 0 | 1 |
| **PD15** | Modeling the Disease Progression from Healthy to Overt Diabetes in ZDSD rats | Semi-mechanistic indirect response models linking insulin, glucose and weight | 36 | cont | 849 | 23 | 37 | 22 | 5 | 0 | 0 | 3 |
| PK: pharmacokinetic; PD: pharmacodynamic; i.v.: intravenous; Ref.: reference; cont: continuous; cat: categorical; N: number; obs: observations; ID: individuals; par: parameters, IIV: inter-individual variability; COV: covariances between variabilities; IOV: : inter-occasion variability; RUV: residual unexplained variability; CMT: compartment; AML: acute myeloid leukaemia. | | | | | | | | | | | | |
