# Supplementary material for: An automated sampling importance resampling procedure for estimating parameter uncertainty
Source: J Pharmacokinet Pharmacodyn. 2017 Sep 8;44(6):509–20. doi: 10.1007/s10928-017-9542-0 (PMC5686280; doi:10.1007/s10928-017-9542-0)
Supplement: Supplementary file 2 — Supplementary material 2 (DOCX 140 kb) [file 10928_2017_9542_MOESM2_ESM.docx]

### Supplementary material 2


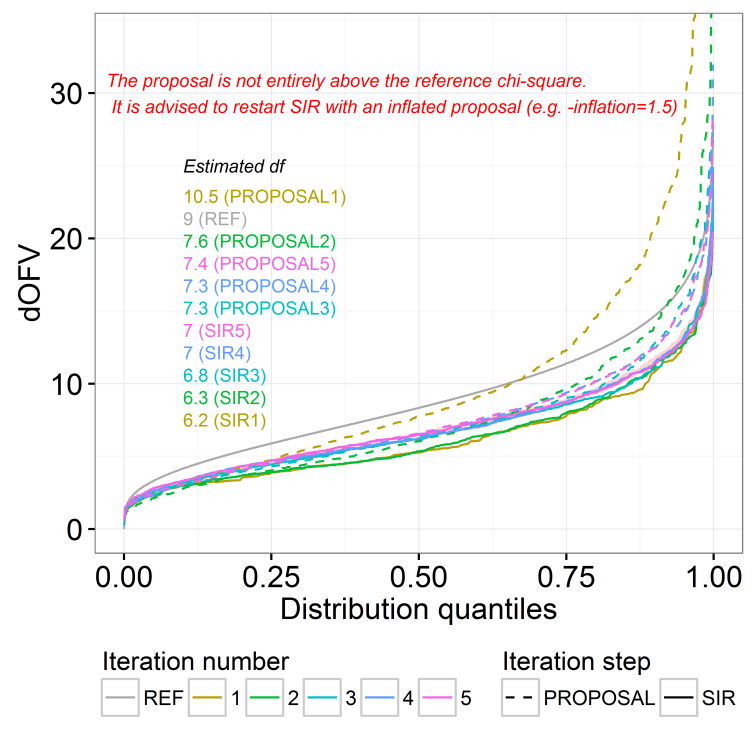


**Figure S1***.* SIR dOFV diagnostic plot showing too narrow proposal distribution; inflation needed (example model PK3).
