# Supplementary material for: An automated sampling importance resampling procedure for estimating parameter uncertainty
Source: J Pharmacokinet Pharmacodyn. 2017 Sep 8;44(6):509–20. doi: 10.1007/s10928-017-9542-0 (PMC5686280; doi:10.1007/s10928-017-9542-0)

### Supplementary material 3

**The example PK5 is taken here to illustrate how SIR should be performed.**

**First, the model (3-compartment PK model with transit absorption and a number of covariate relationships, 18 estimated parameters in total) was run using the PsN command:**

execute Mida_PK_BA1_COV.mod

**Minimization was successful. Estimation and covariance time for this dataset (20 subjects, 808 observations) was 25 minutes. SIR was then performed using the covariance matrix as proposal distribution:**

sir Mida_PK_BA1_COV.mod -dir=sir_cov -rplots=2

SIR took 7 hours to run (note that a bootstrap of the same dataset took 7 hours as well). Diagnostics available in the PsN_plots_base.pdf file looked as follows (the reader is directed to the PSN user guide for a full description of the plots):


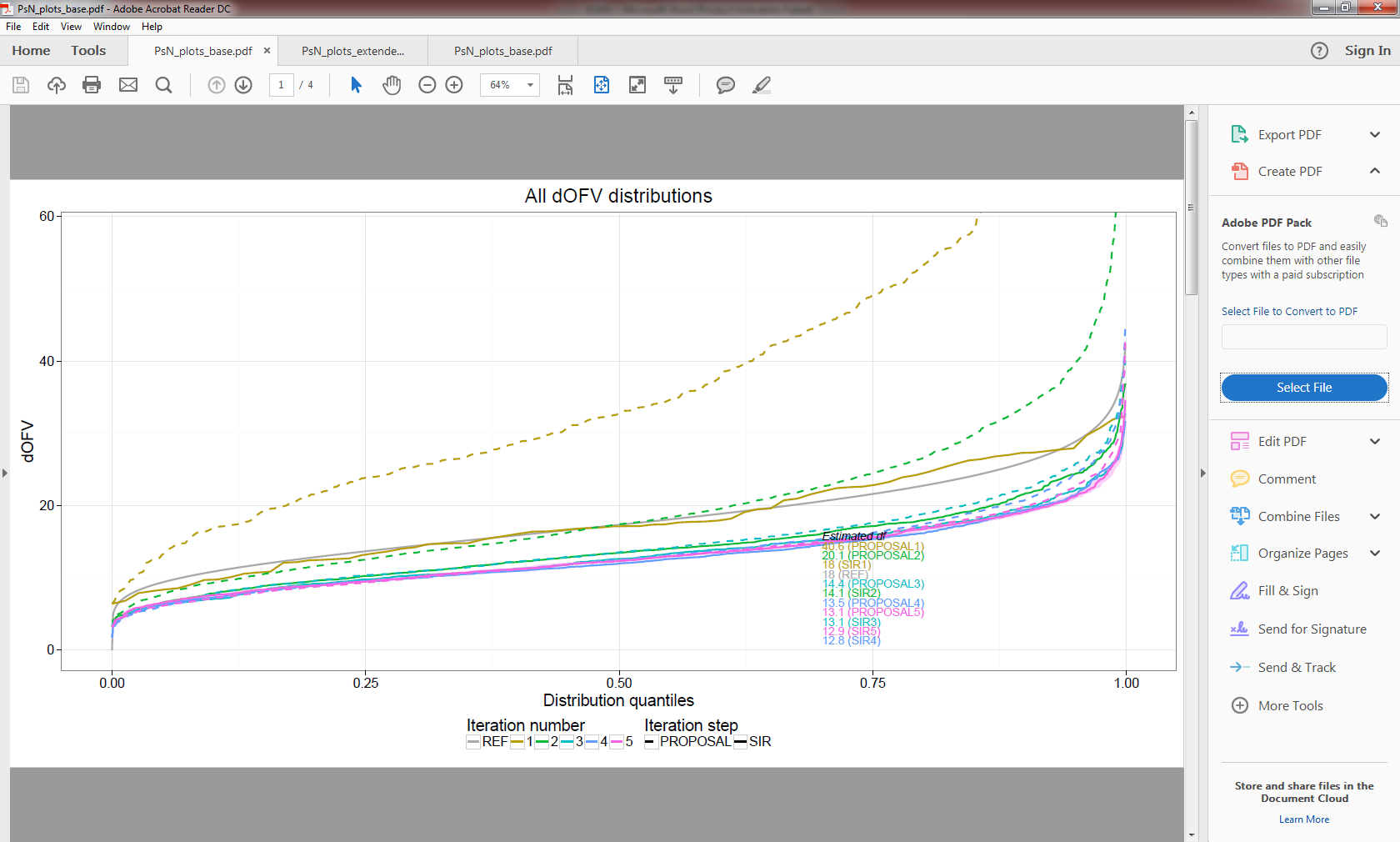


**The dOFV plot showed that the proposal distribution was wide enough (i.e. above the reference chi-square distribution) and that SIR had converged (SIR distribution of iterations 3, 4 and 5 overlaid). SIR results can thus be considered valid.**

**Additional plots show SIR convergence parameter by parameter:**


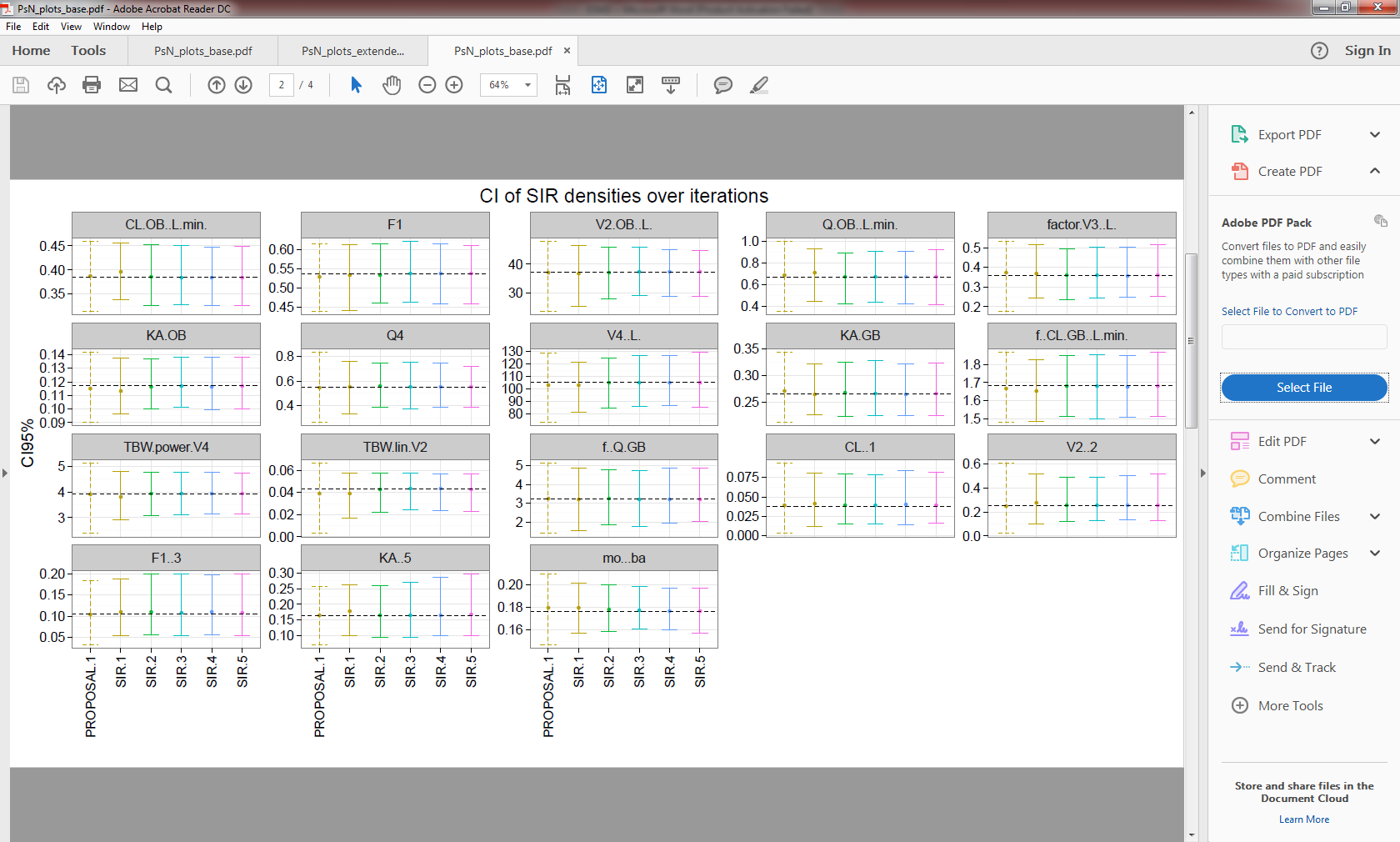


As well as a visual representation of the SIR results summarized as a covariance matrix (note that the same visual representation is also available for the proposal distribution):


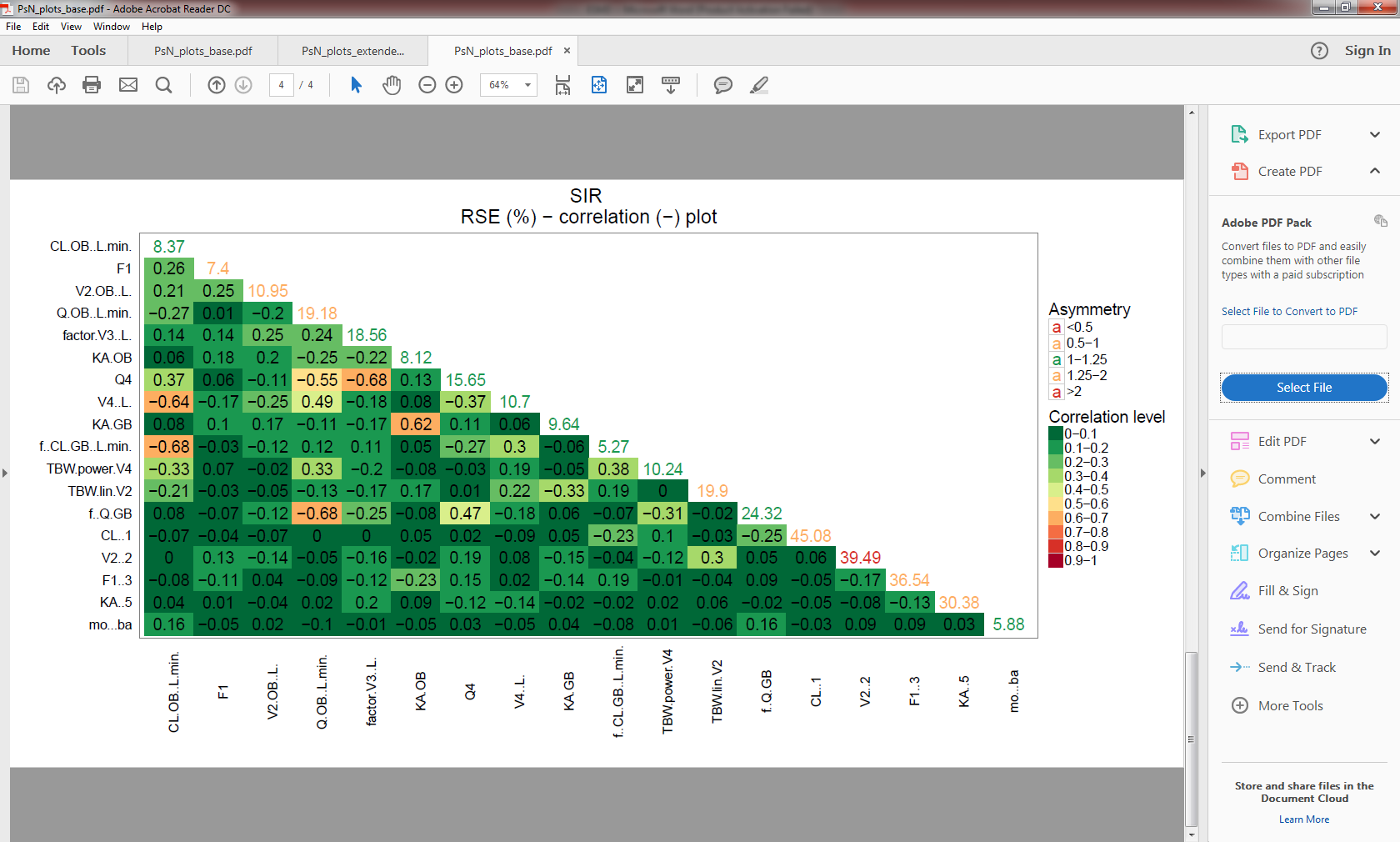

Supplement: Supplementary file 3 — Supplementary material 3 (DOCX 617 kb) [file 10928_2017_9542_MOESM3_ESM.docx]
